# Diets containing traditional and novel green leafy vegetables improve liver fatty acid profiles of spontaneously hypertensive rats

**DOI:** 10.1186/1476-511X-12-168

**Published:** 2013-11-05

**Authors:** Melissa Johnson, Ralphenia D Pace, Norma L Dawkins, Kyle R Willian

**Affiliations:** 1 College of Agriculture, Environment and Nutrition Science, Tuskegee University, Tuskegee, AL 36088, USA; 2 Department of Food and Nutritional Sciences, Tuskegee University, Tuskegee, AL 36088, USA; 3 Department of Chemistry and Biochemistry, Auburn University, Auburn, AL 36849-5312, USA

## Abstract

**Background:**

The consumption of green leafy vegetables (GLVs) has been demonstrated to reduce the risks associated with cardiovascular and other diseases. However, no literature exists that examines the influence of traditional and novel GLVs on the liver fatty acid profile of an animal model genetically predisposed to developing hypertension. The aim of the present study was to examine the effects of diets containing 4% collard greens, purslane or sweet potato greens on the liver fatty acid profiles of four-week old male spontaneously hypertensive rats (SHRs, N = 44). Following four weeks consumption of the diets, liver fatty acid profiles were determined by gas–liquid chromatography of transesterified fatty acid methyl esters.

**Results:**

SHRs consuming the control diet had greater percentages of liver saturated fatty acid and less omega-3 fatty acid percentages. SHRs consuming the diets containing vegetables had significantly greater liver concentrations of γ- linolenic, docosahexaenoic and docosahexaenoic acids, as well as lower levels of lauric, palmitic and arachidonic acids. SHRs consuming the control diet had significantly greater percentages (p < 0.05) of oleic; significantly less γ-linolenic and docosahexaenoic acids.

**Conclusions:**

This study demonstrates the ability of GLVs to modulate liver fatty acid composition, thus providing protection against elevations in atherogenic fatty acids, which may be involved in CVD pathogenesis. Consequently, dietary recommendations for the prevention of CVD should consider the possible cardioprotective benefits and the subsequent alterations in fatty acid profiles afforded by diets containing collard greens, purslane and sweet potato greens.

## Background

Cardiovascular disease (CVD) is the leading cause of morbidity and mortality in the US as well as developing nations [1]. In addition to genetic predisposition, risk factors for CVD include atherogenic dietary patterns affluent in processed food products, total fat, saturated fat, cholesterol, refined carbohydrates and sodium and inadequate intakes of whole grain products, fruits, vegetables and dietary fiber [2-5].

Plants, particularly green leafy vegetables (GLVs), serve as a major dietary reservoir of the essential PUFAs, dietary fiber, antioxidants and other bioactive compounds that exert cardioprotective and chemopreventive biological mechanisms [6-9]. Epidemiological studies have revealed that consumption of fruits and vegetables may be inversely associated with risk for CVD and certain cancers, with the prevalence of disease much lower among individuals consuming near or above the recommended servings compared to those consuming fewer than the recommended intakes [10-12].

Collard greens (*Brassica oleracea*), a traditional GLV consumed mainly in the southern region of the United States, in addition to purslane (*Portulaca oleracea*) and sweet potato greens (*Ipomoea batatas* L.), novel GLVs to the typical American diet, may prove beneficial in reducing the risks associated with CVD development and progression [13-17]. Research has demonstrated reductions in CVD risk following consumption of these green leafy vegetables, both *in vivo* and *in vitro*[18-22]. Although epidemiological evidence has affirmed the association between GLV consumption and reductions in disease risk, there is limited research examining the effects of diets containing collard greens (CG), purslane (PL) and sweet potato greens (SPG) on cardiovascular disease risk, namely liver fatty acid profiles. To test the hypothesis that diets containing CG, PL and SPG (4% dry weight) will contribute to improvements in liver fatty acid profiles the present study was designed to determine the effects of diets containing these GLVs on the liver fatty acid profile of animals genetically predisposed to developing hypertension, the spontaneously hypertensive rat. This study will advance human nutrition research with inferences made regarding the effects of consumption of traditional and novel GLVs on CVD risk factors. Additionally, further recommendations regarding supplementation of these GLVs into the diet on CVD risk may be presented.

## Results

### Liver saturated fatty acids

Liver saturated fatty acid concentrations of SHRs consuming the various diets are presented in Table 1. SHRs consuming the control diet had trace amounts of lauric acid (C12:0) that was significantly (P < 0.05) greater than those consuming the experimental diets. Significantly greater amounts of pentadecanoic acid (C15:0) were present in the livers of SHRs consuming PL diet (0.09 ± 0.01) in comparison to those consuming the SPG diet (0.06 ± 0.01).

**Table 1:** **Liver saturated fatty acid concentrations of SHRs fed diets for 4 weeks**^**§**^

		**Dietary group**			
**Fatty acid**	**Structure**	**Control**	**4% CG**	**4% PL**	**4% SPG**
Capric	C10:0	nd	nd	nd	nd
Undecylic	C11:0	nd	nd	nd	nd
Lauric	C12:0	0.01 ± 0.00^a^	0.00 ± 0.00^b^	0.00 ± 0.00^b^	0.00 ± 0.00^b^
Tridecylic	C13:0	1.60 ± 0.43^a^	1.12 ± 0.25^a^	1.02 ± 0.39^a^	1.33 ± 0.30^a^
Myristic	C14:0	0.29 ± 0.04^a^	0.29 ± 0.06^a^	0.29 ± 0.05^a^	0.26 ± 0.06^a^
Pentadecanoic	C15:0	0.07 ± 0.01^ab^	0.08 ± 0.01^ab^	0.09 ± 0.01^a^	0.06 ± 0.01^b^
Palmitic	C16:0	21.01 ± 0.70^a^	18.44 ± 4.24^a^	19.47 ± 2.67^a^	16.95 ± 3.07^a^
Heptadecanoic	C17:0	0.27 ± 0.13^a^	0.64 ± 0.32^a^	0.78 ± 0.27 ^a^	0.88 ± 0.38^a^
Stearic	C18:0	16.30 ± 0.71^a^	20.71 ± 4.60^a^	17.45 ± 2.53^a^	18.31 ± 2.53^a^
Arachidic	C20:0	0.08 ± 0.04^a^	0.04 ± 0.00^a^	nd	0.05 ± 0.00^a^
Behenic	C22:0	0.11 ± 0.02^a^	0.31 ± 0.08^a^	0.39 ± 0.15^a^	0.40 ± 0.09^a^
Lignoceric	C24:0	0.41 ± 0.16^a^	0.59 ± 0.24^a^	1.23 ± 1.05^a^	0.62 ± 0.38^a^

^§^Data are (expressed as) mean percentage ± SE. Values in the same row that do not share the same superscript letter are significantly different according to analysis of variance and Duncan’s post hoc procedures (P < .05); nd = not detected.

Control = AIN-76 A control diet; 4% CG = AIN-76A diet + 4% collard green powder; 4% purslane = AIN-76A diet + 4% purslane powder; 4% SPG = AIN-76A diet + 4% sweet potato green powder.

### Liver monounsaturated fatty acids

SHRs consuming diets containing CG, PL and SPG exhibited significantly lower liver concentrations of palmitoleic (C16:1n7) acid (0.78 ± 0.19, 0.90 ± 0.14, 0.12 ± 0.02, respectively) than those consuming the control diet (1.42 ± 0.06). Liver oleic acid (C18:1n9) was significantly increased following 4 weeks consumption of the control diet (12.69 ± 1.03). Among SHRs consuming diets containing GLVs, oleic acid concentration was greater with consumption of the PL diet (7.70 ± 1.14), followed by the CG (7.40 ± 1.95) and SPG (5.57 ± 1.16) diets. A more than two-fold significant increase in eicosenoic acid (C20:1n12) was present among SHRs assigned to the PL dietary group (0.28 ± 0.00) in comparison to those assigned to the control group (0.12 ± 0.03) (Table 2).

**Table 2:** **Liver monounsaturated fatty acid concentrations of SHRs fed diets for 4 weeks**^**§**^

		**Dietary group**			
**Fatty acid**	**Structure**	**Control**	**4% CG**	**4% PL**	**4% SPG**
Pentadecenoic	C15:1n5c	0.09 ± 0.02^a^	0.04 ± 0.00^a^	0.02 ± 0.00^a^	0.05 ± 0.00^a^
Palmitelaidic	C16:1 t	0.18 ± 0.03^a^	0.14 ± 0.02^a^	0.15 ± 0.03^a^	0.12 ± 0.02^a^
Palmitoleic	C16:1n7	1.42 ± 0.06^a^	0.78 ± 0.19^b^	0.90 ± 0.14^b^	0.74 ± 0.15^b^
Heptadecenoic	C17:1n7	0.04 ± 0.01^a^	1.56 ± 1.53^a^	0.07 ± 0.03^a^	0.03 ± 0.01^a^
Cis-vaccenic	C18:1n7c	3.36 ± 0.61^a^	2.05 ± 0.31^a^	2.20 ± 0.10^a^	2.33 ± 0.12^a^
Elaidic	C18:1n9t	1.90 ± 0.21^a^	5.95 ± 4.11^a^	1.53 ± 1.43^a^	2.49 ± 2.45^a^
Oleic	C18:1n9c	12.69 ± 1.03^a^	7.40 ± 1.95^b^	7.70 ± 1.14^b^	5.57 ± 1.16^b^
Trans-vaccenic	C18:1n11	1.82 ± 0.00^a^	0.16 ± 0.09^a^	0.65 ± 0.41^a^	1.42 ± 0.00^a^
Eicosenoic	C20:1n12	0.12 ± 0.03^a^	0.16 ± 0.01^ab^	0.28 ± 0.00^b^	0.16 ± 0.06^ab^

^§^Data are (expressed as) mean percentage ± SE. Values in the same row that do not share the same superscript letter are significantly different according to analysis of variance and Duncan’s post hoc procedures (P < .05); nd = not detected.

Control = AIN-76 A control diet; 4% CG = AIN-76A diet + 4% collard green powder; 4% purslane = AIN-76A diet + 4% purslane powder; 4% SPG = AIN-76A diet + 4% sweet potato green powder.

Values in rows with multiple superscript letters (e.g. ab) are not significantly different from other values containing the shared superscript letter (e.g. a or b).

### Liver polyunsaturated fatty acids

SHRs in the control group exhibited significantly lower amounts of γ-linolenic (GLA, C18:3n6) and docosahexaenoic (DHA, C22:6n3) acids versus those consuming the experimental diets containing GLVs (Table 3). GLA percentages ranged from 0.93 ± 0.49 (control) to 5.59 ± 0.73 (PL); DHA percentages ranged from 5.16 ± 0.35 (control) to 8.53 ± 0.63 (SPG). Significantly less docosatrienoic acid (C22:3n3) was present following the consumption of the PL diet (0.60 ± 0.11) versus the control diet (1.01 ± 0.07). A significantly greater amount of docosahexaenoic acid (C22:6n3) was present following the consumption of the experimental diets containing CG (8.31 ± 1.27), PL (8.44 ± 1.02) and SPG (8.53 ± 0.63) in comparison to the control diet (5.16 ± 0.35).

**Table 3:** **Liver polyunsaturated fatty acid concentrations of SHRs fed diets for 4 weeks**^**§**^

		**Dietary group**			
**Fatty acid**	**Structure**	**Control**	**4% CG**	**4% PL**	**4% SPG**
Linolelaidic	C18:2n6t	0.49 ± 0.19^a^	0.09 ± 0.01^a^	0.17 ± 0.05^a^	0.32 ± 0.27^a^
Linoleic	C18:2n6c	14.07 ± 0.68^a^	16.44 ± 0.01^a^	16.45 ± 2.78^a^	16.56 ± 2.82^a^
α-Linolenic	C18:3n3	0.10 ± 0.01^a^	0.10 ± 0.02^a^	0.10 ± 0.01^a^	0.07 ± 0.02^a^
γ-Linolenic	C18:3n6	0.93 ± 0.49^a^	3.70 ± 0.18^b^	5.59 ± 0.73^b^	3.55 ± 1.20^b^
Eicosadienoic	C20:2n6	0.34 ± 0.04^a^	0.27 ± 0.06^a^	0.39 ± 0.09^a^	0.23 ± 0.04^a^
Eicosatrienoic	C20:3n3	0.09 ± 0.02^a^	0.27 ± 0.14^a^	0.18 ± 0.10^a^	0.26 ± 0.15^a^
Dihomo-γ-linolenic	C20:3n6	0.32 ± 0.02^a^	0.31 ± 0.04^a^	0.44 ± 0.03^b^	0.37 ± 0.02^ab^
Arachidonic	C20:4n6	20.39 ± 1.27^a^	14.15 ± 3.44^a^	15.80 ± 3.16^a^	18.51 ± 3.58^a^
Eicosapentaenoic	C20:5n3	0.10 ± 0.02^a^	0.10 ± 0.01^a^	0.19 ± 0.06^a^	0.10 ± 0.01^a^
Docosadienoic	C22:2n6	0.03 ± 0.02^a^	4.09 ± 4.04^a^	0.09 ± 0.04^a^	0.07 ± 0.04^a^
Docosatrienoic	C22:3n3	1.01 ± 0.07^a^	0.76 ± 0.09^ab^	0.60 ± 0.11^b^	0.87 ± 0.11^ab^
Docosatatraenoic	C22:4n6	0.68 ± 0.04^a^	0.64 ± 0.08^a^	0.78 ± 0.11^a^	0.85 ± 0.10^a^
Docosapentaenoic	C22:5n3	1.95 ± 0.42^a^	2.98 ± 0.73^a^	2.34 ± 0.34^a^	1.72 ± 0.19^a^
Docosahexaenoic	C22:6n3	5.16 ± 0.35^a^	8.31 ± 1.27^b^	8.44 ± 1.02^b^	8.53 ± 0.63^b^

^§^Data are (expressed as) mean percentage ± SE. Values in the same row that do not share the same superscript letter are significantly different according to analysis of variance and Duncan’s post hoc procedures (P < .05); nd = not detected. Control = AIN-76 A control diet; 4% CG = AIN-76A diet + 4% collard green powder; 4% purslane = AIN-76A diet + 4% purslane powder; 4% SPG = AIN-76A diet + 4% sweet potato green powder.

Values in rows with multiple superscript letters (e.g. ab) are not significantly different from other values containing the shared superscript letter (e.g. a or b).

### Liver total fatty acids

Although not statistically significant, SHRs consuming the control diet exhibited a greater percentage of liver total saturated fatty acids [23] (39.68 ± 1.51) and a lesser percentage of total unsaturated fatty acids (UFA; 62.18 ± 1.22) (Table 4). SHRs consuming the experimental diets containing CG, PL and SPG demonstrated greater percentages of omega-3 fatty acids (7.88 ± 1.16, 7.67 ± 1.38, 8.62 ± 0.64, respectively) in comparison to those consuming the control diet (5.27 ± 0.34). Omega-3 fatty acid percentages, as well as EPA + DHA percentages were significantly greater following consumption of the SPG diet than the control diet.

**Table 4:** **Total liver fatty acid concentrations and ratios of SHRs fed diets for 4 weeks**^**§**^

	**Dietary group**			
**Fatty acid**	**Control**	**4% CG**	**4% PL**	**4% SPG**
Saturated	39.68 ± 1.51^a^	33.99 ± 7.63^a^	32.57 ± 5.91^a^	33.16 ± 5.64^a^
Unsaturated	62.18 ± 1.22^a^	67.64 ± 7.52^a^	67.84 ± 5.81^a^	68.97 ± 5.03^a^
Monounsaturated	18.25 ± 1.51^a^	20.22 ± 4.58^a^	22.14 ± 5.09^a^	20.17 ± 4.43^a^
Polyunsaturated	43.92 ± 1.51^a^	47.42 ± 6.27^a^	45.70 ± 1.89^a^	48.80 ± 1.04^a^
Omega-3 fatty acids	5.27 ± 0.34^a^	7.88 ± 1.16^ab^	7.67 ± 1.38^ab^	8.62 ± 0.64^b^
Omega-6 fatty acids	34.47 ± 1.47^a^	32.25 ± 2.15^a^	30.59 ± 3.06^a^	35.07 ± 1.05^a^
Omega-6/3 ratio	6.68 ± 0.23^a^	9.05 ± 4.97^a^	8.17 ± 4.87^a^	4.31 ± 0.33^a^
LA/ALA ratio	14.13 ± 1.51^a^	11.95 ±1.89^a^	12.17 ± 1.89^a^	17.48 ± 3.44^a^
EPA + DHA	5.20 ± 0.34^a^	7.82 ± 1.17^ab^	7.62 ± 1.38^ab^	8.59 ± 0.65^b^

^§^Data are (expressed as) mean percentage ± SE. Values in the same row that do not share the same superscript letter are significantly different according to analysis of variance and Duncan’s post hoc procedures (P < .05); nd = not detected. Control = AIN-76 A control diet; 4% CG = AIN-76A diet + 4% collard green powder; 4% purslane = AIN-76A diet + 4% purslane powder; 4% SPG = AIN-76A diet + 4% sweet potato green powder.

Saturated = sum of saturated fatty acids; unsaturated = sum monounsaturated fatty acids + sum polyunsaturated fatty acids); monounsaturated = sum of monounsaturated fatty acids; polyunsaturated = sum of polyunsaturated fatty acids; omega-3 fatty acids = sum of C18:3n3, C20:5n3 and C22:5n3; omega-6 fatty acids = sum of C18:2n6 and C20:4n6; omega-6/3 ratio = omega-6/omega-3 fatty acids; LA/ALA ratio = C18:2n6 (linoleic acid, LA)/C18:3n3 (α-linolenic acid, ALA); EPA + DHA = sum of C20:5n3 (eicosapentaenoic acid, EPA) and C22:6n3 (docosahexaenoic acid, DHA).

Values in rows with multiple superscript letters (e.g. ab) are not significantly different from other values containing the shared superscript letter (e.g. a or b).

## Discussion

Green leafy vegetables such as collard greens, traditionally consumed in the southern region of the US, purslane and sweet potato greens, relatively novel in the American diet, which are rich sources of antioxidants, MUFA, PUFAs and n-3 series PUFAs have demonstrated the ability to attenuate the risks associated with disease pathogenesis [24-29]. In the present study experimental diets containing these GLVs were able to contribute to increased SHR liver concentrations of cardio-protective omega-3 polyunsaturated fatty acids compared to the control diet. Medeiros et al. demonstrated the ability of diets enriched with the omega-3 fatty acid ALA to significantly increase the omega-3 fatty acid (i.e. DHA) content of the rat liver [30]. Although percentages of liver ALA were similar among SHRs consuming the different diets in the present study, greater percentages of DHA were observed among those consuming the diets containing the GLVs; SHRs consuming the PL diet exhibited a greater percentage of EPA compared to those in the other dietary groups. Interestingly, although ALA content was slightly less in the livers of SHRs consuming the SPG diet, DHA content was greater in comparison to those consuming the other diets. Greater percentages of omega-3 fatty acids within the livers of SHRs consuming diets containing GLVs, particularly EPA and DHA, suggest potential cardioprotection and reduced disease risk as demonstrated by others [31].

Greater concentrations of omega-3 PUFAs compared to omega-6 PUFAs among SHRs consuming diets containing the GLVs implies that omega-6 PUFAs may be metabolized to a lesser extent due to the competitive nature of these PUFAs for participation in metabolic pathways. In the current study liver ALA concentrations were similar across all dietary groups, whereas LA concentrations were greater following consumption of the experimental diets containing CG, PL and SPG; liver AA concentrations were increased following consumption of the control diet. The shift towards a trajectory favoring the metabolism of ALA to a greater degree than LA, suggest the ability of diets containing GLVs to promote health and prevent disease.

The data generated from the present study examining the liver fatty acid composition of SHRs consuming diets containing traditional and novel GLVs indicate significant differences in key atherogenic as well as cardioprotective fatty acids. Research has shown an increased risk for CVD with increasing concentrations of the saturated fatty acids lauric, myristic and palmitic, which are considered to be more atherogenic [32]. In the present study, SHRs consuming the control diet demonstrated greater liver concentrations of both lauric and myristic acids. Further, higher percentages of cardioprotective fatty acids of the omega-3 series were found in the livers of SHRs fed diets containing GLVs. The presence of higher levels of these fatty acids suggest potential decreased disease risk via the cardioprotective mechanisms of omega-3 fatty acids generated during anti-inflammatory pathways present during cardiovascular and other disease pathogenesis.

## Conclusions

To our knowledge, this is the first study to examine the effects of diets containing traditional (i.e. collard greens) and novel (i.e. purslane and sweet potato greens) green leafy vegetables on the liver fatty acid profile of the spontaneously hypertensive rat. The current research findings are in agreement with epidemiological evidence, which suggests the cardioprotective and chemopreventive advantages of consuming diets containing dark green leafy vegetables. The results of this research suggest the ability of diets containing collard greens, purslane and sweet potato greens to favourable shift SHR liver fatty acid compositions in such a way that may be beneficial in reducing CVD risk in spontaneously hypertensive rats.

The chief limitation of the current study was the duration of feeding of the control and experimental diets to the spontaneously hypertensive rats. As CVD is a chronic, inflammatory disease occurring over the course of the lifespan, extending the duration of feeding may offer critical insights regarding long-term consumption of novel and traditional GLVs on the pathogenesis of CVD. In addition, focusing on only one aspect of CVD risk, namely the liver fatty acid profile, fails to offer a comprehensive view of the larger spectrum involved in CVD pathogenesis. Examining the effects of diets containing collard greens, purslane and sweet potato greens on factors such as the complete lipid profile, lipid and other nutrient metabolism and other relevant indices of cardiovascular health, such as cardiac hypertrophy, inflammatory biomarkers, tissue morphology, gene expression and SHR physiology, may offer critical evidence regarding the influence of these diets on disease outcomes. Further, implications and translation of the current and future studies to human nutrition and cardiovascular outcomes needs to be explored as well.

In addition, future research studies may want to consider the isolation of nutrient and bioactive compounds within these diets that may have contributed to the presence of a beneficial fatty acid profile. Accordingly, moderate to long-term feeding studies involving the consumption of these diets by SHRs, as well as the consumption of these diets by “normal” rats, not genetically predisposed to developing hypertension warrant further consideration. Therefore, confirming the relationship between shifts in liver fatty acid profile on indices of cardiovascular health or CVD disease pathogenesis, through continued research studies will add credence to the physiological relevance of the current research study.

## Methods

### Animals and diets

Forty-four (N = 44) male spontaneously hypertensive rats (SHRs), approximately 4 weeks of age and weighing 100 grams were obtained from Charles River laboratories (St. Louis, MO). The animals were housed individually in stainless steel cages at the animal care wing of the Tuskegee University Comparative Medicine Research Center. Room temperature and relative humidity were maintained between 68 and 72°C and 55%, respectively. SHRs were maintained on a 12-hour light/12-hour dark cycle.

Experimental diets (Table 5), formulated according to the American Institute of Nutrition (AIN) standards were isocaloric and isonitrogenous, and manufactured by the TestDiet® division of the Purina Mills Company (Richmond, IN). Collard greens (CG) and purslane (PL) were purchased from local farmers markets and sweet potato greens (SPG) were grown on the campus of Tuskegee University. Vegetables were washed, blotted dry and frozen for at least 24 hours prior to freeze drying for approximately 48 hours (Virtis Genesis 25SL freeze-dryer; Virtis Company, Gardiner, NY); following freeze-drying, the samples were ground to pass through a 60-mesh sieve. GLV powders were stored in an air tight container and shipped to the Purina Mills Company for incorporation into the experimental diets. Following subjection to a one week acclimation period, SHRs were randomly assigned to one of four dietary groups (n = 11 per dietary group): AIN-76A purified diet (control), AIN-76A diet containing 4% collard greens (CG; 4% dry weight), AIN-76A diet containing 4% purslane (PL; 4% dry weight) or AIN-76A diet containing 4% sweet potato greens (SPG; 4% dry weight).

**Table 5:** Ingredient composition of control and experimental diets fed to SHRs for 4 weeks

	**Dietary group**			
**Ingredient (g/kg)**	**Control**	**4% CG**	**4% PL**	**4% SPG**
Sucrose	50.0	50.0	48.0	47.0
Casein	20.0	20.0	19.0	19.0
Corn starch	15.0	15.0	15.0	15.0
Powdered cellulose	5.0	2.0	4.0	5.0
Corn oil	5.0	5.0	5.0	5.0
AIN-76 mineral mix	3.5	4.0	4.0	4.0
AIN-76 vitamin mix	1.0	1.0	1.0	1.0
DL-methionine	0.3	0.3	0.3	0.3
Choline bitartrate	0.2	0.2	0.2	0.2
Collard greens	-	4.0	-	-
Purslane	-	-	4.0	-
Sweet potato greens	-	-	-	4.0

Animals were pair fed based on the previous day’s dietary intake of the control group and allowed to consume food and water *ad libitum*. Following the 4-week feeding experiment, animals were fasted overnight and anaesthetized by overinhalation of carbon dioxide, followed by cardiac puncture. SHR livers were removed and stored at −80°C prior to analysis. The procedures involved in the care and use of animals in this research were approved by the Tuskegee University Institutional Animal Care and Use Committee.

### Liver fatty acid analysis

The extraction of total liver fatty acids was conducted using the method of Folch et al. [33]. Briefly, thawed liver tissue samples (~1 g) were homogenized in a chloroform-methanol (2:1 vol/vol) mixture under nitrogen in an ice bath and total lipids extracted. The homogenate was filtered through Whatman No. 4 filter paper and 0.1 M NaCl solution (4:1 ratio) was added to the filtered sample solution. The sample was then flushed with nitrogen, vortexed for 3 minutes and centrifuged at 2,000 rpm for 5 minutes. The organic layer was collected and dried off using a nitrogen evaporator. Extracted total lipids were then transferred into glass tubes and transmethylated by subjection to 1 ml of boron trifluoride (12% in methanol; Fisher Scientific, Fair Lawn, NJ), followed by incubation on a dry heating block at 110 to 115°C for 30 minutes to produce fatty acid methyl esters (FAMEs). The tube was then placed into an ice bath for 5 minutes; 2.0 ml of pentane and 1.0 ml of deionized water was added, the sample was flushed with nitrogen and vortexed for 15 seconds. Following centrifugation for 5 minutes at 2,000 rpm the top layer of the sample was collected and transferred into a pre-weighed 13×100 Pyrex culture tube. Dichloromethane (DCM) was added (3 mL) and the sample was dried using a nitrogen evaporator. Prior to gas chromatography injection, the sample was resuspended in 200 μl of DCM.

Fatty acid methyl esters were isolated and quantified by gas chromatography using a Hewlett-Packard 6890 gas chromatograph containing a fused silica capillary column (DB-23 60.0 m × 250 μm × 0.25 μm) furnished with a flame-ionization detector. A volume of 1 μL of the sample was injected by the auto sampler at a 10:1 split ratio. Helium served as the carrier gas, with a flow rate of 2.0 mL/min. Injector and column temperatures were positioned at 250°C. The initial oven temperature was 130°C and held for 1 minute followed by a 6.5°C/min increment increases in temperature until a temperature of 170°C was achieved. The temperature was again increased at 2.75°C per minute until a temperature of 215°C was reached and maintained for 12 minutes. A final temperature of 230°C was reached and maintained for 3 minutes. Eluted FAMEs were identified based on comparisons to retention times and peaks of known FAMEs contained in an external standard (GLC 463 Standard, NU Check (Elysian, MN)). Areas under peak curves were considered proportional to the amount of the analyte present in the chromatograph. Peak area measurements were obtained and individual FAMEs within each sample were determined as a percentage of total fatty acids present.

### Statistical analysis

Individual liver fatty acids are presented as mean percentage ± standard error (SE) of total fatty acids. One-way analysis of variance and Duncan’s post hoc procedures were conducted to determine if statistically significant differences existed among the different dietary groups. Statistical significance was determined at p < .05. Statistical analyses were performed using Statistical Analysis System (SAS) software (SAS, Inc. Cary, NC).

## Competing interests

The authors declare that they have no competing interests.

## Authors’ contributions

MJ performed the animal study, liver fatty acid profile analysis, analyzed data, and drafted the manuscript. RDP contributed to the conception and design of the study, supervised the project and edited the manuscript. NLD contributed to the design of the study and edited the manuscript. KRW supervised the fatty acid profile analysis and edited the manuscript. All authors read and approved the final manuscript.
